# Enhancer of Zeste Homolog 2 Inhibition Attenuates Atrial Fibrillation Post-Myocardial Infarction Through Reduced Atrial Fibrosis

**DOI:** 10.31083/RCM51343

**Published:** 2026-09-01

**Authors:** Tian-Peng Wei, Wen-Rui Wang, Xiao-Lu Zhang, Huan-Huan Liu, Ying Liu, Fan Yang, Xiao-Yu Liu, Ru-Xing Wang, Ling-Ling Qian

**Affiliations:** ^1^Department of Cardiology, Wuxi People’s Hospital Affiliated to Nanjing Medical University, 214023 Wuxi, Jiangsu, China

**Keywords:** enhancer of zeste homolog 2, atrial fibrillation, myocardial infarction, fibrosis, arrhythmia

## Abstract

**Background:**

Atrial fibrillation (AF) is one of the most prevalent arrhythmias following a myocardial infarction (MI), yet the pathogenesis associated with post-MI AF remains poorly understood. Enhancer of zeste homolog 2 (EZH2), an enzyme that regulates histone modifications, has been implicated in various cardiovascular diseases; however, the role of EZH2 in the development of post-MI AF also remains unclear.

**Methods:**

MI was induced in mice by ligation of the left anterior descending coronary artery (LAD), followed by intraperitoneal administration of GSK126 or vehicle control (polyethylene glycol 300 and saline).

**Results:**

MI significantly increased the incidence and duration of AF, while electrical mapping revealed decreased conduction velocity. These changes were accompanied by increased atrial fibrosis, which was associated with upregulation of EZH2 and trimethylated histone H3 at lysine 27 (H3K27me3). In GSK126-treated mice, the incidence of AF was reduced, along with a significant decrease in H3K27me3 expression and atrial fibrosis.

**Conclusion:**

Upregulation of EZH2 is associated with post-MI AF. Pharmacological inhibition of EZH2 using GSK126 attenuates atrial fibrosis and reduces the incidence of AF. These findings suggest that EZH2 may represent a potential therapeutic candidate for post-MI AF, although further validation is required.

## 1. Introduction

Myocardial infarction (MI), a major cardiovascular disease of global concern, significantly increases the risk of atrial fibrillation (AF), with an incidence ranging from approximately 4% to 21% among MI patients, and this rate is gradually rising [1]. More importantly, post-MI AF substantially increases the risk of adverse outcomes, such as heart failure and stroke, which significantly alter a patient’s prognosis [2,3]. However, the pathogenesis of post-MI AF remains incompletely understood, which limits the development of clinical treatment regimens. Therefore, exploring the underlying mechanisms of AF following an MI is of great importance for clinical prevention and treatment.

Currently, epigenetic modifications are closely associated with the development of numerous diseases. Classic research indicates that these modifications include DNA methylation, histone methylation, and histone acetylation, among others [4]. Notably, histone methylation has been implicated in the progression of various cardiovascular diseases, including myocardial fibrosis [5,6]. This modification is mediated by histone methyltransferases, which play a crucial role in regulating gene expression [4]. Enhancer of Zeste Homolog 2 (EZH2), a well-characterized histone methyltransferase, has been demonstrated to participate in the process of atrial fibrosis and AF [7]. However, there is a lack of research regarding its influence on the incidence of AF in patients following an MI. Therefore, the present study aims to investigate the impact of EZH2 on the development of post-MI AF.

## 2. Materials and Methods

### 2.1 Animal Ethics

Adult male C57BL/6 mice, aged 6 weeks, were procured from Changzhou Cavens Laboratory Animal Co., Ltd. All animal experiments adhered to the guidelines established by the Chinese Academy of Health and Animal Husbandry and received approval from the Ethics Committee of Wuxi People’s Hospital Affiliated to Nanjing Medical University. The mice were housed in a specific pathogen-free environment with controlled humidity, temperature, and a light cycle of 12 hours of light followed by 12 hours of darkness. They were provided with standard mouse food and water ad libitum.

### 2.2 Mice MI Model Construction and Euthanasia

The 7-day model of MI in mice was established via left anterior descending coronary artery (LAD) ligation. Briefly, anesthesia was induced via intraperitoneal injection of tribromoethanol (150 mg/kg), followed by tracheal intubation. A midline sternotomy was performed to expose the third intercostal space, and the LAD was ligated using an 8-0 suture. Successful induction of myocardial ischemia was confirmed by the appearance of blanching in the anterior wall of the left ventricle. After evacuating intrathoracic blood and air, the muscles and skin were sutured in layers. The sham group underwent the same surgical procedures, with the exception of LAD ligation [8].

In the MI + GSK126 group, the EZH2 inhibitor GSK126 was dissolved in a vehicle solution composed of polyethylene glycol 300 and saline and administered via intraperitoneal injection following MI modeling. For the vehicle control group (MI + Vehicle), an equivalent volume of the polyethylene glycol 300 and saline vehicle, devoid of GSK126, was injected using the same method. GSK126, obtained from HY-13470, MedChemExpress, New Jersey, USA, was administered intraperitoneally to mice at a dosage of 30 mg/kg after LAD ligation. The drug was injected once a day for a total of 7 days [7]. The investigator who administered the treatments was not involved in data collection or analysis, and all assessments were blinded to group allocation until completion.

Following the completion of data collection, the mice were placed in an anesthesia induction chamber and continuously exposed to an inhalational overdose of isoflurane. The oxygen flow rate was maintained at 0.5 L/min, while the isoflurane concentration was adjusted to 5% and sustained until the mice achieved deep anesthesia and respiratory arrest. Subsequently, cervical dislocation was performed to ensure euthanasia. All procedures were reviewed and approved by the Institutional Animal Care and Use Committee (Multi-channel small animal anesthesia machine, RWD, China; Isoflurane, Orbiepharm, China).

### 2.3 Echocardiography

A week following the myocardial infarction, an echocardiogram was performed to evaluate myocardial function and the size of the left atrium in the mice. The mice were given mild anesthesia with 1.5%–2% isoflurane during the procedure. The echocardiographic assessments utilized the Vevo 2100 system (Visual Sonics, Canada), and the data analysis was performed using M-mode or two-dimensional echocardiography.

### 2.4 Electrocardiography

Mice were sedated using an intraperitoneal injection of tribromoethanol (150 mg/kg). To induce AF, a solution containing acetylcholine (25 μg/mL) and calcium chloride (6 mg/mL) was administered through the tail vein at a dosage of 10 mL/kg [9]. This model evaluates AF inducibility rather than spontaneous AF. AF was defined as an irregular RR interval with no discernible P wave, lasting at least 1 second. All Electrocardiograms (ECG) were analyzed by two independent investigators who were blinded to group allocation. ECG data were collected using a biological signal acquisition system (BL-420N, China) for subsequent analysis. All induction agents were obtained from Sigma, USA.

### 2.5 Masson’s Trichrome Staining (Masson Staining)

For the Masson staining procedure, paraffin sections underwent a series of dewaxing and dehydration steps using xylene and graded ethanol solutions, followed by a rinse in tap water. A three-color staining solution from Servicebio (G1006-100ML, China) was utilized. The sections were soaked in Masson A solution overnight and then washed. Next, they were stained for one minute with a 1:1 blend of Masson B and C solutions, rinsed, and briefly differentiated in acid alcohol. After washing, the sections were treated with Masson D solution for six minutes, rinsed again, and stained with Masson E for one minute. The slides were then placed directly into Masson F for thirty seconds, followed by differentiation in 1% acetic acid. Dehydration was achieved through two changes of absolute ethanol, and clearing was performed using xylene. Finally, the sections were mounted with neutral resin and observed under a light microscope to capture the images. All chemicals were obtained from Servicebio.

To quantify the degree of fibrosis, a transverse section from each left atrium was analyzed. The anatomical sample region was the left atrial (LA) free wall, and perivascular areas were manually excluded [10]. Images were captured at 20× magnification. Collagen volume fraction was quantified using ImageJ software (version 1.54, National Institutes of Health, Bethesda, MD, USA) with a fixed threshold applied consistently across all images by an investigator blinded to group allocation, calculated as (blue area / total tissue area) × 100%.

### 2.6 Triphenyltetrazolium Chloride Staining

The heart was gently rinsed with cold PBS at 4 °C, then patted dry and frozen at –20 °C for 10 minutes. The heart was then embedded in a heart matrix and sliced into sections. The sections were incubated in a 2,3,5-triphenyltetrazolium chloride (TTC) solution (17779, Millipore, USA) in the dark at 37 °C for 20 minutes. After staining, the sections were washed three times with cold phosphate-buffered saline (PBS, G4202-500 mL, Servicebio, China), with each wash lasting 5 minutes. The sections were then fixed in paraformaldehyde overnight, rinsed with cold PBS, and photographed. The red regions indicated healthy myocardium, while the lighter areas showed infarcted myocardium. The infarct size was quantified using ImageJ and calculated as: (Total pale area across all sections / Total area of all sections) × 100%.

### 2.7 Electrical Mapping

Mice were sedated using intraperitoneal injections of tribromoethanol (150 mg/kg) and treated with heparin sodium (3.125 U/kg) through an intraperitoneal injection [11]. This was followed by retrograde perfusion of the heart with Krebs-Henseleit (KH) solution, which contained the following concentrations in mM: 119 NaCl, 25 NaHCO_3_, 10 D-glucose, 4 KCl, 1.2 KH_2_PO_4_, 1 MgCl_2_·6H_2_O, and 1 CaCl_2_·2H_2_O, all maintained at 37 °C with a constant flow rate of 2.5 mL/min.

A 36‑electrode array (PA03606060302, Sigao Institute of Electrophysiology, China) was gently placed on the left atrial surface. Atrial electrograms were recorded during sinus rhythm (no external pacing) using EMapScope software (version 5.941, MappingLab, Shenzhen, China). Activation times were manually annotated at each electrode using the maximum negative dV/dt, with the earliest activation site as the reference time. Conduction velocity was calculated from activation maps using the median value (mm/ms). Conduction inhomogeneity (dispersion) was defined as the absolute value of activation time variation across adjacent electrodes (ms/mm). Data were collected using EMapScope software (version 5.941), followed by analyses to assess the conduction velocity and heterogeneity within the left atrium. All analyses were performed by an investigator blinded to the group allocation.

### 2.8 Western Blot

Proteins were isolated from the LA tissues of mice utilizing RIPA lysis buffer (89901, Thermo Fisher Scientific, USA). Following this, the proteins underwent separation through sodium dodecyl sulfate-polyacrylamide gel electrophoresis (SDS-PAGE) and were subsequently transferred to polyvinylidene fluoride membranes (03010040001, Millipore, USA). The membranes were then treated with a blocking solution of 5% non-fat milk and incubated with primary antibodies overnight at 4 °C. Secondary antibodies were then applied, and the protein bands were detected using an enhanced chemiluminescence reagent (Millipore, Billerica, MA, USA).

The antibodies utilized in this study comprised the following: Enhancer of Zeste Homolog 2 (EZH2, 5246S, CST, USA), trimethylated Histone H3 at Lysine 27 (H3K27me3, 9733S, CST, USA), Histone 3 (H3, 9715S, CST, USA), collagen I (14695-1-AP, Proteintech, USA), Transforming Growth Factor-beta1 (TGF-β1, 81746-2-RR, Proteintech, USA), Alpha-Smooth Muscle Actin (α-SMA, 14395-1-AP, Proteintech, USA), and Glyceraldehyde-3-Phosphate Dehydrogenase (GAPDH, 60004-1-lg, Proteintech, USA). For secondary antibodies, we employed Peroxidase-AffiniPure Goat Anti-Mouse IgG (H + L) (115-035-003, Jackson, USA) and Horseradish Peroxidase-conjugated Goat Anti-Rabbit IgG (H + L) (A0208, Beyotime, China).

### 2.9 RNA Extraction and Quantitative Real-Time PCR

LA tissues were processed using Trizol reagent (Thermofisher, 10296010CN) in nuclease-free microcentrifuge tubes. A fifth of the volume of chloroform was added to the mixture, which was then vigorously vortexed and centrifuged. The resulting upper aqueous layer was carefully transferred to a fresh tube and combined with an equal amount of isopropanol to facilitate RNA precipitation. After centrifugation, the liquid above the RNA pellet was discarded, and the pellet was rinsed with 75% ethanol. Following another centrifugation, the ethanol was removed, and the pellet was left to air-dry until it became translucent before being reconstituted in nuclease-free water.

The concentration of RNA was measured with a microvolume spectrophotometer (NanoDrop™ One/OneC, Thermofisher, USA). Reverse transcription was carried out in accordance with the guidelines provided by the reverse transcription kit (HiScript III RT SuperMix for qPCR (+ gDNA wiper), Vazyme, R323-01, China) utilizing a thermal cycler (MiniAmp™ Plus, Thermofisher, USA). Subsequently, quantitative real-time PCR was performed based on the instructions of the qPCR detection kit (ChamQ Blue Universal SYBR qPCR Master Mix, Vazyme, Q312-02, China) using a thermal cycler (SimpliAmp™, ABI, USA). The associated PCR primers are listed in Table 1. Relative mRNA expression levels were calculated using the 2^−ΔΔCt^ method. *Gapdh* was used as the endogenous reference gene. qPCR cycling conditions: Initial denaturation at 95 °C for 30 s; followed by 40 cycles of 95 °C for 10 s, 60 °C for 30 s. Melting curve: 95 °C for 15 s, 60 °C for 60 s, gradual ramp to 95 °C.

**Table 1.** **Primer sequences used for qRT-PCR**.

Gene (mice)	Forward primer	Reverse primer
*Ezh2*	5′-ACCACAGGATAGGCATCT-3′	5′-GGGAAGAGGTAGTAGATGTCAAG-3′
*Gapdh*	5′-AAGAAGGTGGTGAAGCAGG-3′	5′-GAAGGTGGAAGAGTGGGAGT-3′

*Ezh2*, enhancer of zeste homolog 2; *Gapdh*, glyceraldehyde-3-phosphate dehydrogenase.

### 2.10 Statistical Analysis

The data analysis was conducted utilizing GraphPad Prism version 8.0 (GraphPad Software, San Diego, CA, USA). Results are expressed as the mean ± standard error. The Shapiro-Wilk test was used to evaluate the normality of the data distribution. For comparing two samples, a two-tailed unpaired Student’s *t*-test was utilized. The normality and homogeneity of variance were verified before the *t*-test. In cases where the normality assumption was not met, the Mann-Whitney U test, which is a non-parametric approach, was used. Statistical significance was set at *p *< 0.05.

## 3. Results

### 3.1 MI Promoted AF Susceptibility

To explore how MI affects the occurrence of AF, we created a mouse model by occluding the LAD. During the procedure, the ECG displayed significant ST-segment elevation immediately after the LAD was occluded, signifying acute myocardial ischemia (Fig. 1A). A week following the MI, we employed TTC staining, which confirmed the model by revealing a markedly larger infarct size in the MI group compared to the control group (Fig. 1B,C). In addition, echocardiography indicated both functional and structural changes in the MI mice, with significant reductions in left ventricular ejection fraction and fractional shortening, indicating compromised ventricular systolic function (Table 2).

**Fig. 1.**
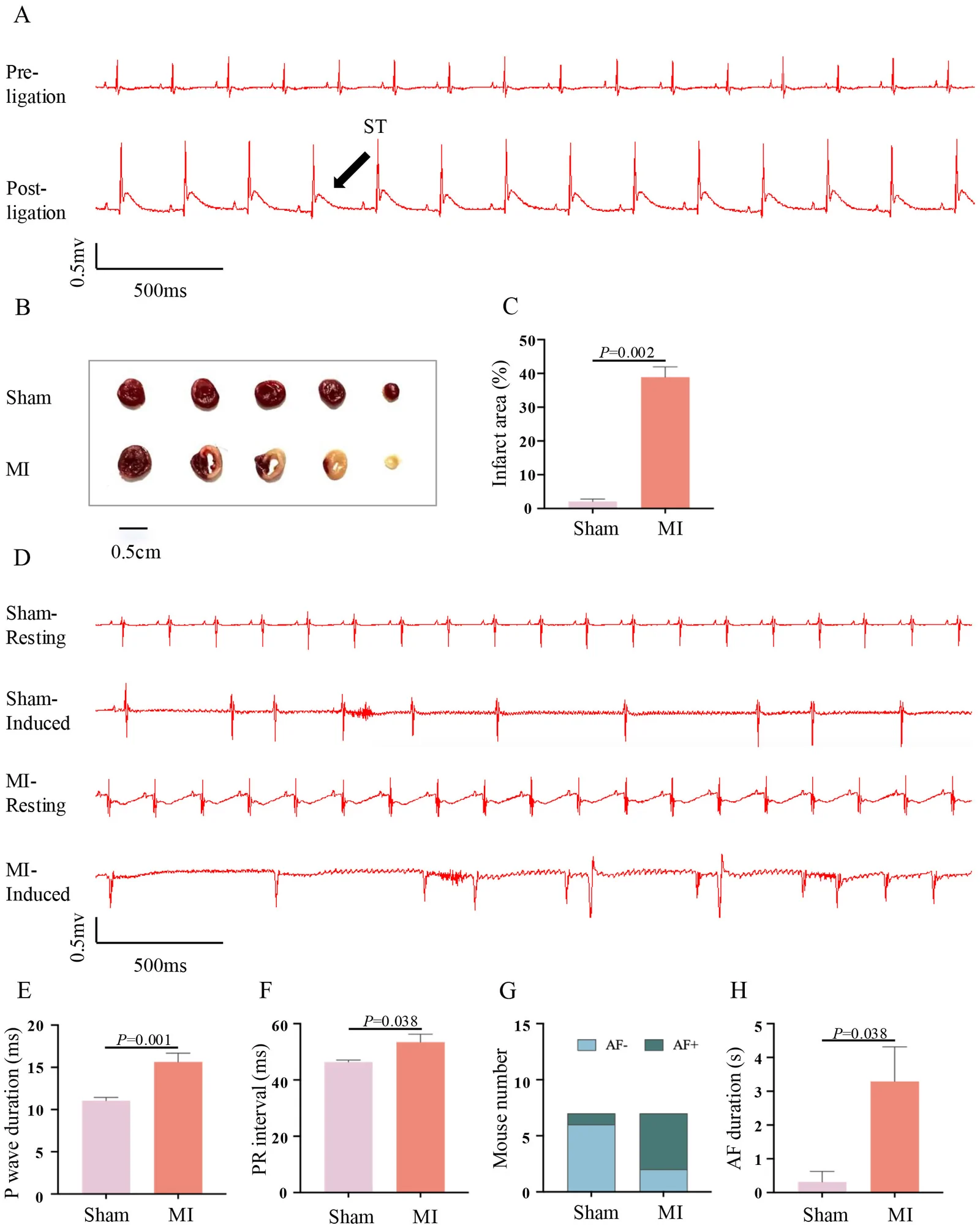
**MI exacerbates atrial remodeling and susceptibility to AF**. (A) Surface ECG of mice in the MI model before and after LAD ligation; the arrow indicates the ST segment. (B) Representative TTC staining of transverse cardiac sections from mice in the Sham and MI groups. (C) Quantitative analysis of infarct size via TTC staining in the Sham and MI groups (*n* = 6). (D) Representative ECG of mice in the Sham and MI groups under resting state and AF-induced state. (E–H) Quantitative analysis of P wave duration (E) and PR interval (F) under resting state, and comparison of AF induction rate (G) and AF duration (H) under AF-induced state in the two groups (*n* = 7). Sham, Sham operation group; MI, myocardial infarction group; LAD, left anterior descending coronary artery; TTC, 2,3,5-triphenyltetrazolium chloride; AF, atrial fibrillation; ECG, Electrocardiogram. Values are displayed as mean ± standard error of the mean, except for (G) where values are *n*.

**Table 2.** **Echocardiographic parameters in Sham and MI groups**.

	Sham	MI	t value	*p* value
LVEF (%)	65.033 ± 0.746	47.255 ± 2.005	8.253	<0.001
LVFS (%)	34.630 ± 0.600	23.426 ± 1.126	8.777	<0.001
LVIDd (mm)	3.297 ± 0.089	4.035 ± 0.136	4.522	<0.001
LVIDs (mm)	2.152 ± 0.048	3.100 ± 0.151	5.968	<0.001
IVSd (mm)	0.735 ± 0.007	0.777 ± 0.027	1.458	0.178
IVSs (mm)	1.121 ± 0.020	1.121 ± 0.040	0.014	0.989

Values are displayed as mean ± standard error of the mean. Sham, The wild-type Sham operation group; MI, Myocardial infarction group; LVEF, Left Ventricular Ejection Fraction; LVFS, Left Ventricular Fractional Shortening; LVIDd, Left Ventricular Internal Diameter at End-Diastole; LVIDs, Left Ventricular Internal Diameter at End-Systole; IVSd, Interventricular Septal Thickness at End-Diastole; IVSs, Interventricular Septal Thickness at End-Systole. *n* = 9.

The vulnerability to drug-triggered AF was also evaluated. Representative ECG recordings of the induced AF are presented (Fig. 1D). When contrasted with the Sham group, mice with an MI showed a significant increase in both P-wave duration and PR interval at rest. During the AF induction trials, the MI cohort revealed a greater tendency for AF induction and a significantly extended duration of AF, suggesting a heightened susceptibility to AF (Fig. 1E–H).

### 3.2 MI-Induced Abnormal Atrial Electrical Conduction Remodeling

The specific mechanisms driving AF post-MI are incompletely understood [12]. Important questions remain regarding whether atrial remodeling after MI represents adaptive or detrimental changes. Accordingly, we analyzed the LA in MI mice. Electrical mapping indicated that MI mice exhibited significantly reduced atrial conduction velocity and increased conduction heterogeneity, suggesting impaired atrial electrical conduction and a potentially increased risk of arrhythmias (Fig. 2A–C). In addition, echocardiographic assessments revealed significant dilation of the left atrium, reflecting structural remodeling in MI mice (Fig. 2D,E). Furthermore, there was a marked increase in the LA weight of these mice (Fig. 2F,G). These combined electrophysiological and structural changes may create an environment conducive to the onset and persistence of AF.

**Fig. 2.**
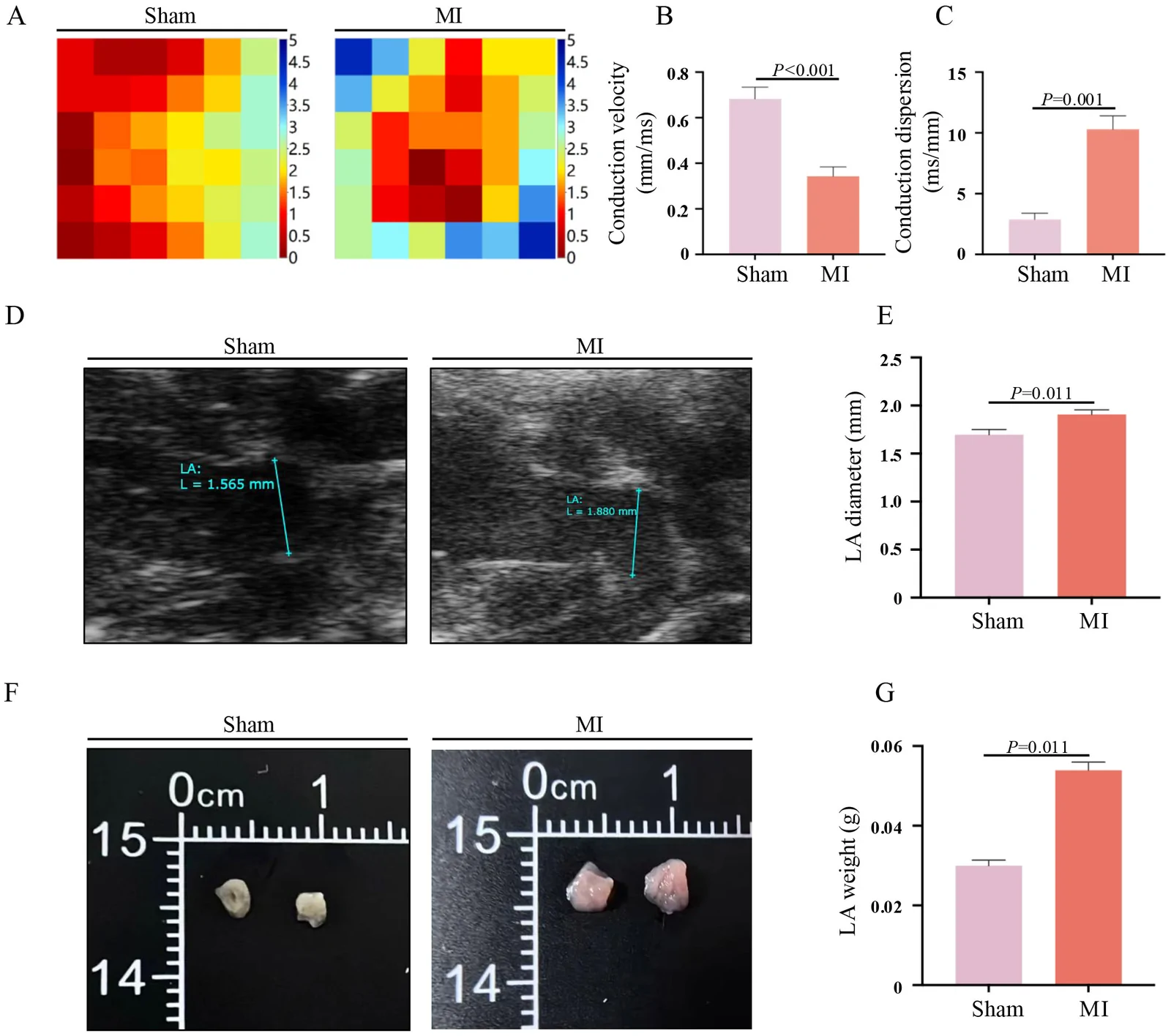
**MI aggravates atrial remodeling**. (A) Representative conduction maps of multi-channel electrophysiological mapping of the LA in mice from the Sham and MI groups. (B,C) Quantitative analysis of LA conduction velocity (B) and conduction inhomogeneity (C) in multi-channel electrophysiological mapping of the two groups (*n* = 5). (D) Representative echocardiograms of the LA obtained from apical four-chamber views in mice of the Sham and MI groups. (E) Quantitative analysis of LA diameter in the Sham and MI groups (*n* = 9). (F) Visual images of atria from mice in the Sham and MI groups. (G) Quantitative analysis of LA weight in the Sham and MI groups (*n* = 6). Sham, Sham operation group; MI, myocardial infarction group; LA, left atrium. Values are displayed as mean ± standard error of the mean.

### 3.3 MI-Induced Atrial Fibrosis and Upregulation of EZH2 Expression

To investigate the structural factors connecting an MI to the risk of AF, we examined atrial fibrosis. Seven days after the MI, we evaluated atrial tissue fibrosis using Masson staining. Our quantitative assessment indicated a significant rise in the proportion of fibrotic area relative to the overall atrial tissue area in mice with an MI (Fig. 3A,B). In line with these findings, Western blot analysis showed an increase in the protein levels of collagen I, Transforming Growth Factor-beta1 (TGF-β1), and α-Smooth Muscle Actin (α-SMA) in the atrial tissues of the MI group (Fig. 3C–E).

**Fig. 3.**
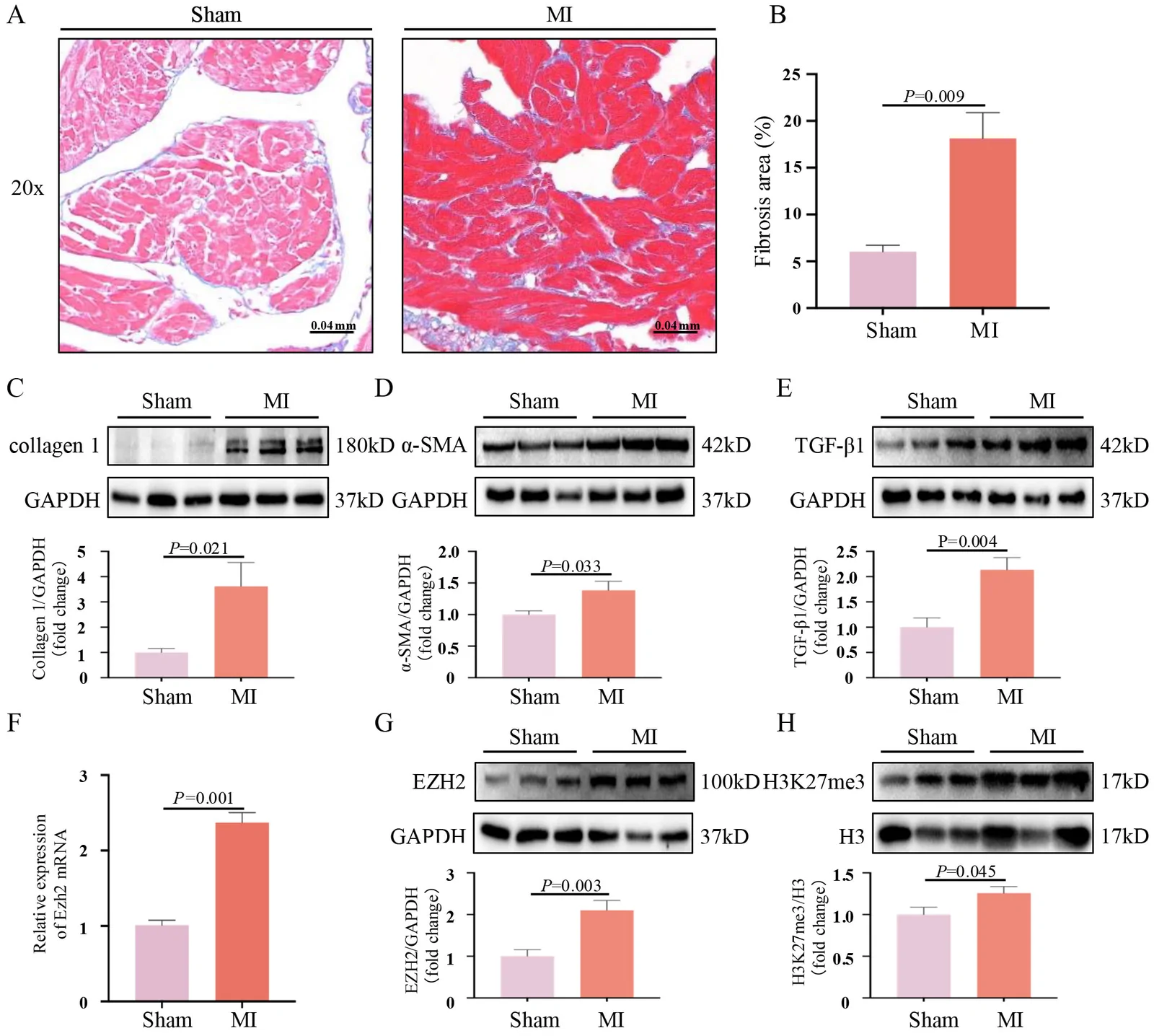
**MI induces atrial fibrosis and upregulates the EZH2/H3K27me3 signaling in mice**. (A,B) Representative Masson staining images (A) and quantitative analysis (B) of atrial fibrosis in the sham and MI groups (*n* = 5). Scale bar = 0.04 mm under 20× field of view. (C–E) Western blot analysis and quantitative analysis of the expression levels of collagen I, α-SMA, and TGF-β1 in the atria of mice from the Sham and MI groups (*n* = 6). (F) qPCR analysis of the expression levels of *Ezh2* mRNA in the atria of mice from the Sham and MI groups (*n* = 6). (G,H) Western blot analysis and quantitative analysis of the expression levels of EZH2 (*n* = 6) and H3K27me3 (*n* = 9) in the atria of mice from the Sham and MI groups. Sham, Sham operation group; MI, myocardial infarction group; α-SMA, alpha-smooth muscle actin; TGF-β1, transforming growth factor-beta1; EZH2, Enhancer of Zeste Homolog 2; H3K27me3, trimethylated Histone H3 at Lysine 27. Values are displayed as mean ± standard error of the mean.

EZH2, the key catalytic component of the Polycomb Repressive Complex 2 (PRC2), plays a crucial role in gene silencing by facilitating the trimethylation of Histone H3 at Lysine 27 (H3K27me3). Abnormal levels of EZH2 have been shown to increase cardiac fibrosis by influencing both the standard and alternative signaling pathways of TGF-β1 [13]. Nevertheless, the precise involvement of EZH2 in post-MI atrial fibrosis is not fully understood. To investigate its role following an MI, we analyzed the expression of EZH2 and H3K27me3 in mouse atrial tissues. Our findings indicated that, compared with the Sham group, both the mRNA and protein levels of EZH2, as well as the protein level of H3K27me3, were significantly elevated in atrial tissues after MI in mice (Fig. 3F–H).

### 3.4 EZH2 Inhibition Reduced AF Susceptibility in MI Mice

The competitive inhibitor of S-adenosylmethionine binds to the active site of EZH2, thereby abrogating EZH2-mediated methylation of H3K27 [14]. Currently, it serves as a canonical inhibitor of EZH2 in basic research [7,15]. Having confirmed that GSK126 successfully suppressed EZH2 catalytic activity via assessment of H3K27me3 levels in the treatment group (Fig. 4A–C), we next analyzed the susceptibility to AF in mice post-MI with the treatment of GSK126.

**Fig. 4.**
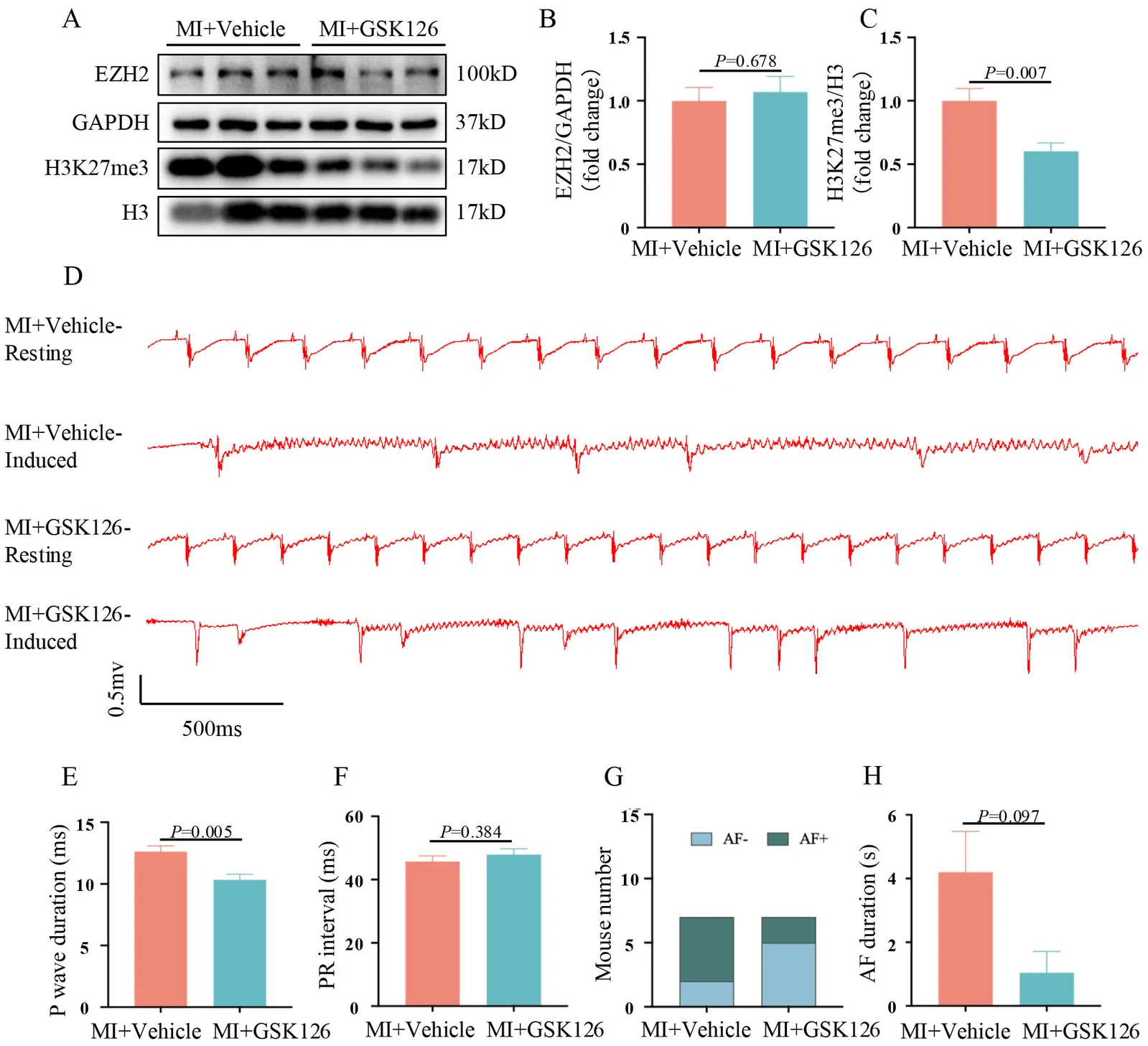
**EZH2 inhibitor GSK126 ameliorates post-MI AF**. (A–C) Western blot analysis and quantitative analysis of the expression levels of EZH2 and H3K27me3 in the atria of mice from the MI + Vehicle and MI + GSK126 groups (*n* = 6). (D) Representative ECG of mice in the MI + Vehicle and MI + GSK126 groups under resting state and AF-induced state. (E–H) Quantitative analysis of P wave duration (E) and PR interval (F) under resting state, and comparison of AF induction rate (G) and duration time (H) under AF-induced state in the two groups (*n* = 7). MI + Vehicle, myocardial infarction with vehicle control group; MI + GSK126, myocardial infarction group treated with GSK126; EZH2, Enhancer of Zeste Homolog 2; H3K27me3, trimethylated Histone H3 at Lysine 27. Values are displayed as mean ± standard error of the mean, except for (G) where values are *n*.

We present the typical ECG and analyzed the susceptibility to AF and the impairment of electrical conduction in the MI + Vehicle and MI + GSK126 groups (Fig. 4D). GSK126 significantly decreased the P-wave duration in MI mice at rest, while the PR interval remained unchanged. The MI + GSK126 cohort showed a lower frequency and shorter duration of AF episodes (Fig. 4E–H). These findings highlight the therapeutic potential of GSK126 and imply that its protective role against AF following MI is facilitated by the inhibition of EZH2.

### 3.5 EZH2 Inhibition Improved Atrial Remodeling

To explore the atrial modifications associated with the decreased vulnerability to AF after GSK126 administration, we conducted additional assessments of its impact on atrial remodeling in mice following an MI. Our electrical mapping indicated that GSK126 contributed to a partial recovery of atrial conduction velocity and reduced conduction heterogeneity after an MI (Fig. 5A–C). Nevertheless, echocardiographic evaluations of atrial size and the statistical analysis of atrial mass did not reveal any significant alterations; however, cardiac function had recovered (Fig. 5D–G and Table 3). In conclusion, these findings suggest that GSK126 offers a partial improvement in abnormal electrical conduction within the atria after an MI.

**Table 3.** **Echocardiographic parameters in MI + Vehicle and MI + GSK126 groups**.

	MI + Vehicle	MI + GSK126	t value	*p* value
LVEF (%)	41.041 ± 2.693	52.438 ± 2.050	3.434	0.004
LVFS (%)	19.695 ± 1.462	26.491 ± 1.221	3.593	0.030
LVIDd (mm)	3.669 ± 0.143	3.797 ± 0.153	0.593	0.563
LVIDs (mm)	2.950 ± 0.136	2.795 ± 0.130	0.815	0.492
IVSd (mm)	0.753 ± 0.022	0.802 ± 0.011	2.044	0.060
IVSs (mm)	1.001 ± 0.046	1.230 ± 0.036	3.972	0.001

Values are displayed as mean ± standard error of the mean. MI + Vehicle, myocardial infarction with vehicle control group; MI + GSK126, myocardial infarction group treated with GSK126; LVEF, Left Ventricular Ejection Fraction; LVFS, Left Ventricular Fractional Shortening; LVIDd, Left Ventricular Internal Diameter at End-Diastole; LVIDs, Left Ventricular Internal Diameter at End-Systole; IVSd, Interventricular Septal Thickness at End-Diastole; IVSs, Interventricular Septal Thickness at End-Systole; LAD, left anterior descending coronary artery. *n* = 7–9.

**Fig. 5.**
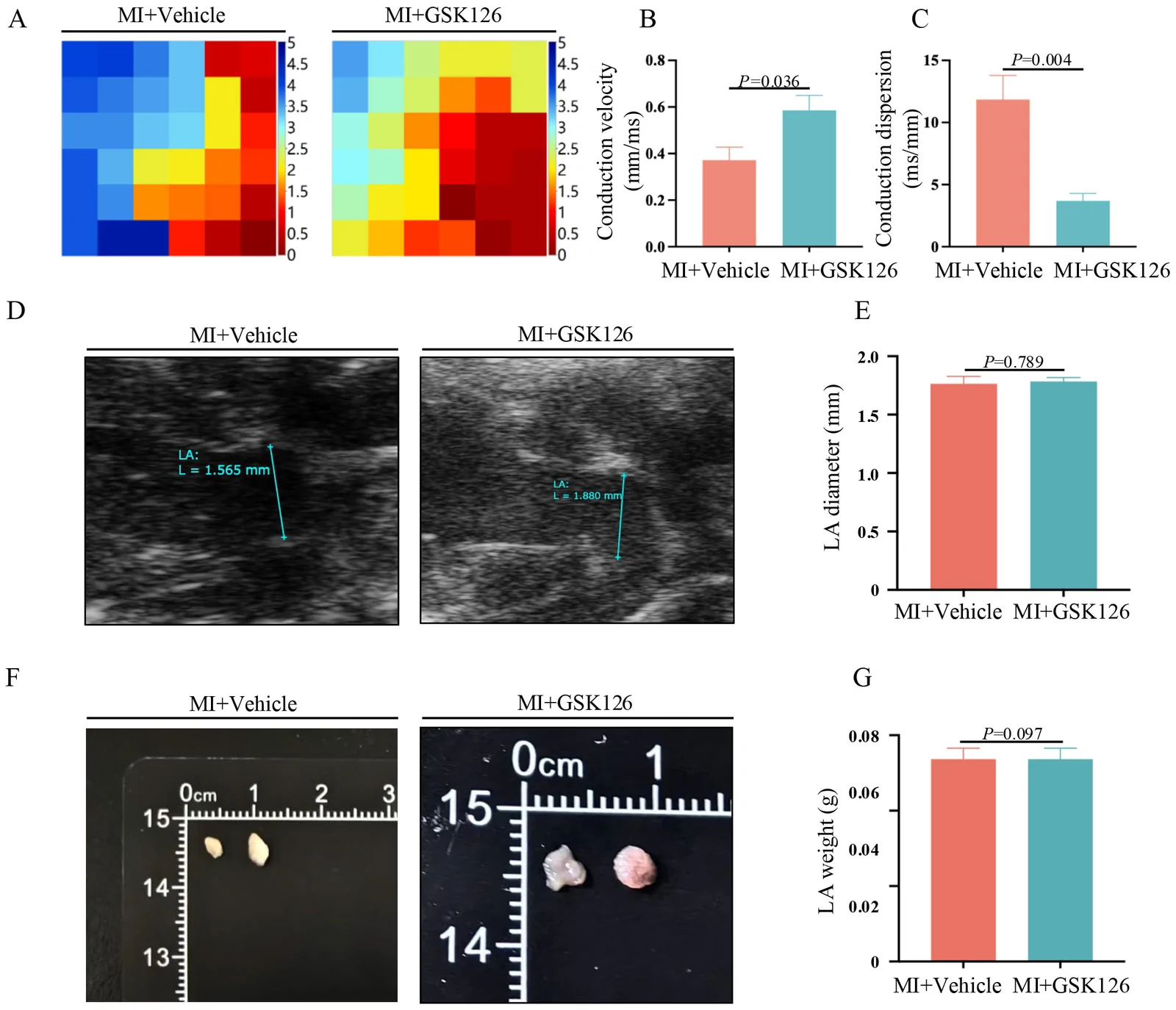
**GSK126 improves post-MI atrial remodeling**. (A) Representative conduction maps of multi-channel electrophysiological mapping of the LA in mice from the MI + Vehicle and MI + GSK126. (B,C) Quantitative analysis of LA conduction velocity (B) and conduction inhomogeneity (C) in multi-channel electrophysiological mapping of the two groups (*n* = 5). (D) Representative echocardiograms of the LA obtained from apical four-chamber views in mice of the MI + Vehicle and MI + GSK126 groups. (E) Quantitative analysis of LA diameter in the MI + Vehicle and MI + GSK126 groups (*n* = 7–9). (F) Visual images of atria from mice in the MI + Vehicle and MI + GSK126 groups. (G) Quantitative analysis of LA weight in the MI + Vehicle and MI + GSK126 (*n* = 6). MI + Vehicle, myocardial infarction with vehicle control group; MI + GSK126, myocardial infarction group treated with GSK126; LA, left atrium. Values are displayed as mean ± standard error of the mean.

### 3.6 EZH2 Inhibition Attenuates Atrial Fibrosis Post-MI AF

To evaluate the impact of GSK126 on atrial fibrosis following an MI, we conducted an analysis of collagen accumulation in atrial tissues using Masson’s trichrome staining. The findings revealed that treatment with GSK126 significantly reduced the extent of atrial fibrosis in mice after an MI (Fig. 6A,B). In addition, there was a significant decrease in the levels of fibrosis-associated markers such as collagen I, TGF-β1, and α-SMA (Fig. 6C–E). These results suggest that GSK126 can effectively minimize the fibrotic changes in the left atrium after an MI, consistent with its influence on critical fibrotic signaling pathways.

**Fig. 6.**
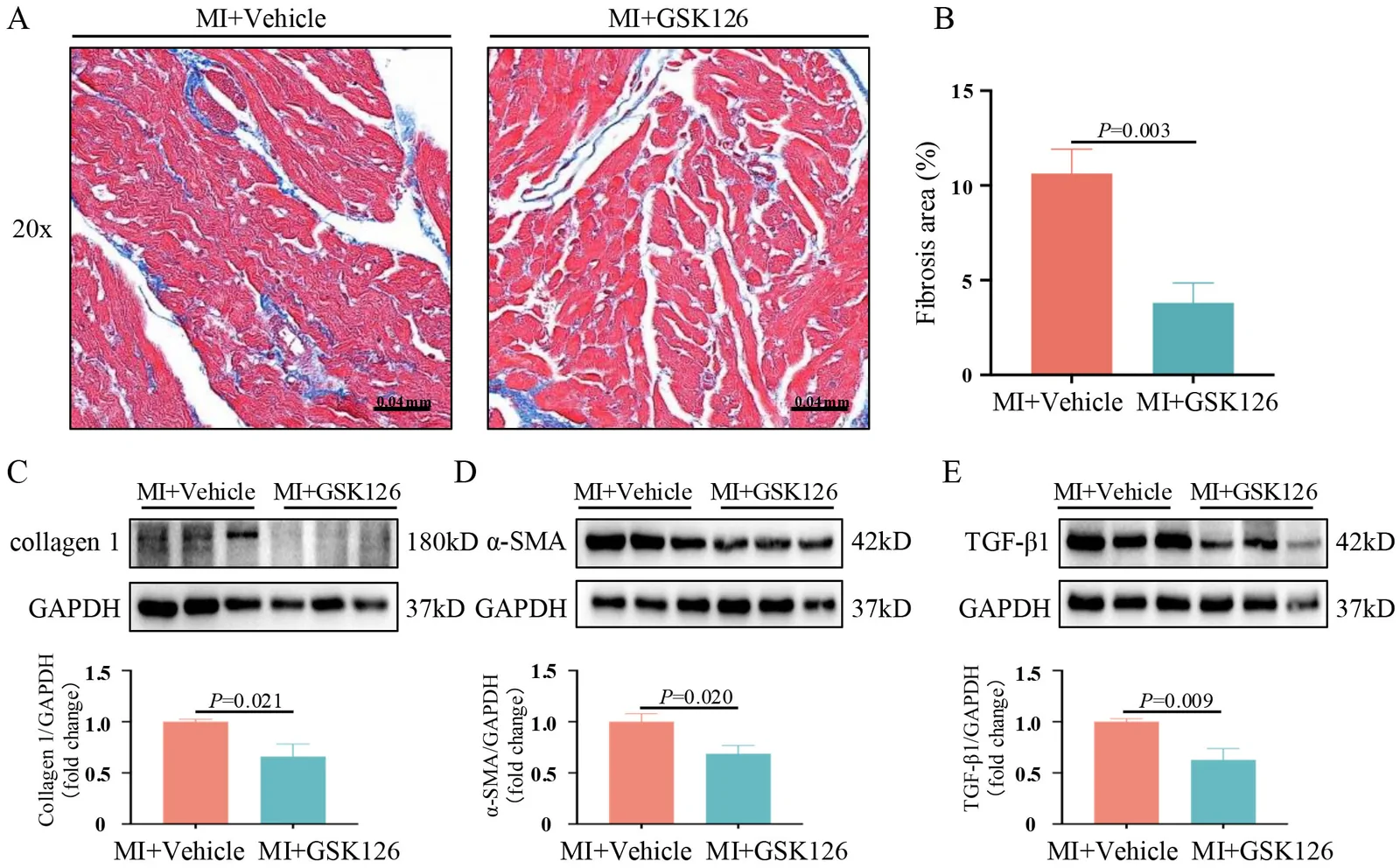
**Inhibition of EZH2 attenuates atrial fibrosis post-MI**. (A,B) Representative Masson staining images (A) and quantitative analysis (B) of atrial fibrosis in the MI + Vehicle and MI + GSK126 groups (*n* = 5). Scale bar = 0.04 mm under 20× field of view. (C–E) Western blot analysis and quantitative analysis of the expression levels of collagen I, α-SMA, and TGF-β1 in the atria of mice from the MI + Vehicle and MI + GSK126 groups (*n* = 6). MI + Vehicle, myocardial infarction with vehicle control group; MI + GSK126, myocardial infarction group treated with GSK126; EZH2, Enhancer of Zeste Homolog 2; α-SMA, alpha-smooth muscle actin; TGF-β1, transforming growth factor-beta1. Values are displayed as mean ± standard error of the mean.

## 4. Discussion

The onset of AF following a MI significantly increases the incidence of heart failure, stroke, and mortality in patients. Nevertheless, the exact molecular pathways involved are not fully understood, leading to a lack of focused treatment options. The main observations were: (1) MI results in atrial fibrosis and a significant increase in the risk of AF, along with an increased expression of EZH2; (2) administration of the EZH2 inhibitor GSK126 successfully reduced atrial fibrosis after an MI, enhanced atrial conduction, and lowered the risk of AF; and (3) EZH2 could serve as a promising target for therapies aimed at post-MI AF.

### 4.1 MI-Induced Atrial Structural and Electrical Remodeling: Relevance to AF Pathogenesis

In our study, we first confirmed that our MI model significantly promoted the formation of an AF substrate, manifested as atrial dilation and fibrosis, along with slowed conduction velocity and increased conduction heterogeneity [16]. These changes strongly supported the central role of “ventricular-atrial crosstalk” in AF pathogenesis [12].

The inflammatory response following an MI plays a crucial role in electrical remodeling, while the changes in the myocardial microenvironment due to atrial inflammation can establish conditions favorable for atrial fibrillation [17]. In addition, chronic hypoxia in the atria, triggered by myocardial ischemia, can further facilitate atrial remodeling [18]. Ventricular dysfunction after an MI may influence the atria through factors such as mechanical stretching and neurohumoral changes, thereby increasing the likelihood of AF.

In our research, we observed that structural changes, particularly fibrosis, and alterations in electrical activity occur simultaneously, indicating a possible causal link. The fibrotic tissue in the atria may interfere with regular electrical conduction, setting the stage for reentrant excitation and potentially leading to atrial fibrillation following a myocardial infarction.

### 4.2 Activation of the EZH2/H3K27me3 Signaling Pathway During Post-MI Atrial Remodeling

Epigenetic control plays a vital role in the processes associated with atrial fibrillation [19,20]. EZH2, a key epigenetic modulator, is a significant contributor to the epigenetic disturbances triggered by ischemia. EZH2 regulates diverse biological processes, such as cell proliferation**, **protein homeostasis, and cardiac development [21,22,23]. Multiple studies indicated that EZH2 was involved in the pathological process and regulates repair following myocardial ischemia/infarction through diverse mechanisms, including modulating apoptosis-related proteins, epigenetically regulating ion channel expression, and influencing immune cell functions [24,25,26,27].

Previous studies confirmed that EZH2 plays a significant role in organ fibrosis and regulates myocardial fibrosis under pathological conditions such as pressure overload and diabetes [28,29]. However, its role in post-MI atrial fibrosis remains unreported. To investigate the role of EZH2 in post-MI AF, we established a murine model of MI. Experiments revealed that at 7 days post-MI, mice exhibited a significantly increased susceptibility to induced AF, accompanied by marked atrial fibrosis. In addition, we observed significantly elevated expression levels of both EZH2 and its catalyzed key histone modification, H3K27me3, in atrial tissues. These results indicate that the EZH2/H3K27me3 signaling pathway is specifically activated during atrial fibrosis and electrical remodeling in mice post-MI, which may represent a key mechanistic link connecting myocardial ischemia, epigenetic alterations, and the onset of AF.

### 4.3 Reduction of AF Susceptibility and Electrophysiological Remodeling Through GSK126

To establish a causal relationship between EZH2 function and post-MI AF, we administered GSK126, a highly selective catalytic inhibitor of EZH2, as an interventional treatment [14]. Multiple research teams have utilized the selective EZH2 inhibitor GSK126 to investigate its role in organ fibrosis across different systems. Specifically, Xia et al. [30] applied GSK126 to inhibit EZH2-mediated macrophage-to-myofibroblast transition (MMT), thereby attenuating the progression of renal fibrosis. Li et al. [31] employed GSK126 to study the function of EZH2 in diabetic myocardial fibrosis, and Song et al. [7] used the same inhibitor to decrease angiotensin II-induced atrial fibrosis.

Our results demonstrated that GSK126 not only significantly reduced H3K27me3 levels in atrial tissue but also partially reversed the atrial electrophysiological impairment induced by MI. More importantly, inhibition of EZH2 produced multiple beneficial effects: it significantly attenuated atrial fibrosis, enhanced intra-atrial conduction velocity, reduced conduction heterogeneity, which translated into reduced AF inducibility and duration. These improvements in epigenetic modification and organ function establish the methyltransferase activity of EZH2 as a critical mechanistic driver of post-MI atrial fibrosis and AF pathogenesis.

Atrial cardiomyopathy is characterized by a constellation of structural, electrical, and functional abnormalities in the atria, which are primarily driven by excessive fibrosis, persistent inflammation, and chronic hemodynamic overload [19,32]. Our present findings support the premise that upregulation of EZH2 contributes to atrial fibrosis and increases vulnerability to atrial fibrillation after a myocardial infarction. Notably, EZH2-mediated H3K27me3 does not function as an isolated signaling event; instead, it likely acts as an important epigenetic regulator within the integrated fibrotic–genomic network of the atrium. By cross-talking with other pro-fibrotic and pro-remodeling pathways, EZH2 may orchestrate multiple pathological processes that collectively promote atrial structural remodeling and the subsequent initiation and maintenance of atrial fibrillation.

While GSK126 intervention significantly ameliorated atrial fibrosis and electrical conduction properties, it did not reverse the MI-induced enlargement of the left atrial internal dimension or the increase in atrial weight. Furthermore, although P-wave duration was partially restored in MI mice, no significant change in PR interval was observed. These findings suggest that the EZH2/H3K27me3 pathway primarily contributes to the more plastic and dynamic “fibrotic” component of post-MI atrial remodeling (e.g., activation of the renin-angiotensin-aldosterone system, enhanced oxidative stress—known pro-fibrotic mechanisms [13]), rather than the acute atrial myocardial stretch and hypertrophy directly driven by early hemodynamic pressure overload [16]. This may be attributed to the fact that atrioventricular conduction is primarily influenced by other mechanisms, such as direct ischemic effects, and is therefore less responsive to GSK126 [33].

The post-MI atrial remodeling process is extensive, and the limited duration and scope of GSK126 intervention may explain why significant reversal of structural remodeling was not observed within this short-term timeframe. This finding carries important clinical implications: it suggests that therapeutic strategies targeting EZH2 may be more suitable for improving the atrial electrophysiological substrate following MI to prevent AF initiation, rather than aiming to reverse established structural dilation of the atria.

## 5. Limitations

This study focused on a 7-day post-MI period in male mice, and AF was induced pharmacologically rather than monitored spontaneously. While our data support a role for EZH2 in atrial fibrosis, the lack of atrial-specific genetic loss-of-function experiments means that causality remains to be firmly established. Future studies using cell-type-specific *Ezh2* knockout and longer observation periods could further define the direct atrial contribution of EZH2, as well as explore downstream transcriptional targets that link EZH2 to atrial fibrosis and arrhythmogenesis.

## 6. Conclusions

Pharmacological inhibition of EZH2 attenuates post-MI AF in mice via suppression of atrial fibrosis. The EZH2 inhibitor GSK126 reduces AF susceptibility and implicates EZH2 in the modulation of post-MI atrial remodeling. Further genetic validation and cell-type-specific studies are still required to clarify causal relationships. At present, EZH2 may represent a plausible therapeutic candidate rather than a fully validated target for mitigating post-MI AF burden.

## Data Availability

All data supporting the conclusions of this study are presented in the main text. Raw data are available from the corresponding author upon reasonable request.
